# The First Observation of Turbulence in Northwestern China by a Near-Space High-Resolution Balloon Sensor

**DOI:** 10.3390/s20030677

**Published:** 2020-01-26

**Authors:** Yang He, Zheng Sheng, Mingyuan He

**Affiliations:** 1 College of Meteorology and Oceanography, National University of Defense Technology, Changsha 410073, China; heyang12357@sina.com (Y.H.); hmy008@sina.com (M.H.); 2 Collaborative Innovation Center on Forecast and Evaluation of Meteorological Disasters, Nanjing University of Information Science and Technology, Nanjing 210094, China

**Keywords:** instrument noise, turbulence, Thorpe analysis, high-resolution balloon sensor

## Abstract

Based on a new type of sensor mounted on a near-space balloon released in Hami, Xinjiang, the Thorpe method was used to analyze turbulence. The method was applied for the first time to northwest China (the mid-latitude region), and almost no radiosonde data above 40 km have been used to study turbulence hitherto. The feasibility of analyzing turbulence characteristics using radiosonde data based on the Beidou positioning system by the Thorpe method was thus verified. The distribution characteristics of turbulence scale, turbulence intensity, and turbulence kinetic energy dissipation rate, and the turbulence diffusion coefficient, were analyzed and discussed. The relationship between turbulence fraction, turbulence intensity, and stratified instability was also investigated. The results show that over 35 km, the influence of instrument noise on turbulence detection is significantly enhanced, which lead to an overestimation of turbulence in that region. The turbulence fraction was defined to reflect the degree of turbulence internal mixing, which is closely related to atmospheric instability. It was found that when the turbulence fraction reached 60%–80%, the turbulence reached its strongest intensity, and when the turbulence fraction exceeded 80%, the turbulence could not be maintained and began to decay.

## 1. Introduction

Turbulence is an important form of atmospheric motion. As one of the causes of atmospheric disturbance, the generation of turbulence can make originally regularly stratified atmospheric fluid produce short-term and high-frequency irregular motion, through which the transmission of mass and energy in the horizontal and vertical directions is significantly enhanced. The physical characteristics of the atmosphere, such as temperature, pressure, and density, are affected by turbulence disturbances, whose values will experience random deviations and irregular fluctuations. Therefore, the meteorological elements reflecting the physical characteristics of the atmosphere can be used as tracers for atmospheric turbulence movement. The causes of atmospheric turbulence mainly include wind shear, thermal convection, latent heat release, and the breakage of gravity waves. These factors, acting alone or together, make the turbulence either fragmentary patches or disturbance layers with a certain thicknesses and strengths. Due to the high intermittence and randomness of turbulence, the corresponding turbulence parameters constantly change. How to detect turbulence more accurately is of great significance to better understanding the transmission mechanism of turbulence to atmospheric material energy and the distribution and variation of turbulence parameters, and to improve the accuracy of numerical weather prediction and climate models.

Due to the wide distribution of turbulent scales, a vortex scale can be as large as hundreds of meters or even kilometers, or as small as a few millimeters. According to the Kolmogorov energy cascade dissipation theory, under the condition of a high Reynolds number, energy will be transferred from a large-scale vortex to a small-scale vortex through the mechanism of inertia. Finally, kinetic energy is dissipated at a very small scale, and kinetic energy is converted into internal energy. The movement of the particles inside the turbulent layer is extremely irregular, so the structure of the turbulence is extremely complicated. At present, the detection methods of atmospheric turbulence are still relatively limited. High-resolution density and wind field data can be obtained by rocket sounding to conduct a spectral analysis and extract turbulence information [1,2]. The turbulence structure can also be observed by radar echoes [3,4,5], and several methods have been developed by previous studies. The radar echo method has a large detection area and good continuity. Also, it is possible to characterize turbulence at high resolutions using remote sensing instruments, such as Doppler wind lidar [6]. However, due to the complex factors affecting the radar echo, the result of the radar echo method is uncertain. The spectrum analysis method can retrieve turbulence more accurately, but it requires an extremely high data resolution, which is only applicable to rocket and balloon detection data with extremely high resolution [7,8]. Further, due to the small number of observation stations, it is difficult for the data to possess the corresponding conditions for turbulence observation by lidar.

Thorpe [9] proposed a method to estimate the overturn scale of turbulent mixing in the ocean and determine the turbulent mixing layer. Due to the commonality of atmospheric and marine fluids, this method was later applied to the analysis and study of sounding balloon data [10,11], therein obtaining good effects for turbulence detection in the atmosphere. This study provided a new idea for the analysis of atmospheric turbulence using conventional radiosonde data with a relatively low vertical resolution. Continuous sounding data obtained from many stations around the world can be used to extract the temporal and spatial distribution of turbulence by the Thorpe method [12,13,14,15]. Meanwhile, the turbulence detected from balloon data can provide a comparative reference for radar and other means [16,17]. Wilson et al. [18] applied Thorpe analysis to data with both a high resolution (10–20 cm) and a low resolution (5–9 m) in the same area and found that the vertical resolution significantly affected the amount and scale of the detected turbulence. 

In previous studies, the height of the sounding data analyzed by the Thorpe method generally reached about 30 km, but verification on the feasibility of using the Thorpe method at a higher height is lacking. In this study, for the first time, the Thorpe method was applied to near-space, high-resolution large-balloon data from the CW-Beidou upper-air meteorological detection system in northwest China. This type of balloon can detect the turbulence in the lower layer of the near space, reaching an altitude above 40 km. Consequently, the results of the turbulence detection from our balloon sounding can cover higher altitudes.

## 2. Materials and Methods

### 2.1. Sensor Introduction and Data Processing

**Figure 1:**
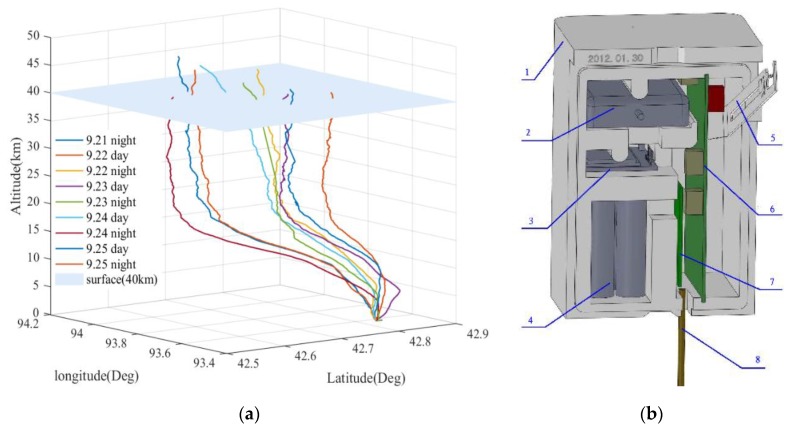
(**a**) A total of nine balloon ascending trajectories from September 21 to September 25, 2019. (**b**) Schematic diagram of the GTS3 radiosonde 1—foam box; 2—Beidou receiving antenna; 3—Beidou module; 4—battery; 5—temperature humidity sensor bracket; 6—smart converter; 7—digital transmitter; and 8—transmitter antenna.

Our team released a total of nine near-space balloons in the Hami area of Xinjiang and obtained radiosonde data from September 21 to September 25 in 2019. The rising trajectory of the balloons is shown in Figure 1a. All balloons reached an altitude of more than 40 km. Among them, the maximum balloon blast height was 45.47 km, and the minimum balloon blast height was 40.26 km. All the sensors carried on the balloon stopped collecting data because of the balloon blast. Based on the CW-Beidou upper-air meteorological detection system, the balloon carried GTS3 radiosonde (Figure 1b), which uses a new type of TPU (temperature-pressure-humidity) sensor to measure atmospheric temperature, humidity, and air pressure, and uses Beidou positioning technology to measure atmospheric wind direction and speed. The beaded thermistor and the wet-sensitive capacitor are welded to the temperature humidity sensor bracket. The air pressure sensor and temperature compensation sensor are mounted directly on the smart converter. The detection accuracy of this system is significantly improved compared with the existing detection systems in China. Its anti-jamming ability, the cost of its ground receiving system, its degree of automation, and its requirements for launching conditions are much better than those of other high-altitude detection systems. The measuring range and accuracy of the instrument are shown in Table 1. The average ascent rate of the radiosonde is 7 m/s (the range of ascent rate during the whole process is between 4 and 13 m/s), and the sampling frequency is 1 Hz.

Due to the influence of instrument noise, the pressure data obtained from the measurement do not decrease monotonically with the height. We adopted the least square cubic spline approximation method [18] to solve the above problem of pressure fluctuation and obtained the pressure data decreasing monotonically with height. The potential temperature, meridional wind, and zonal wind were calculated by using existing data. After the quality control of each element, the re-interpolation was carried out on a uniform grid with a vertical step size of 7 m, and the resampled data were used as the analysis data for this paper.

### 2.2. Turbulence Retrieval Method

The Thorpe method determines the inversion region of potential temperature by comparing the original potential temperature profile with the reordered profile. Particles on the original potential temperature profile move up or down adiabatically so that the sorted potential temperature increases monotonically with the height and reaches a state of minimum total energy. At this point, the height difference before and after the sorting of each particle is defined as Thorpe displacement [19]. For a complete turbulent patch, the sum of the internal Thorpe displacement is 0; that is:

(1) $$\left\{ \begin{array}{l} \sum_{i=m}^{i=k}D_{T}(i)=0,(k=n)\\ \sum_{i=m}^{i=k}D_{T}(i)<0,(m<k<n) \end{array}\right.$$

where m is the lower boundary of the turbulent patch, and n is the upper boundary of the turbulent patch. All inversion regions of the whole profile can be determined by these criteria. The Thorpe length $L_{T}$ is the root mean square of the Thorpe displacement within a turbulent patch; i.e., $L_{T}=rms(D_{T})$. For an independent turbulent layer, Thorpe displacement with a larger overall distribution level can give a larger Thorpe length. In this way, the Thorpe length can reflect the turbulence intensity. The thickness of the turbulent layer can be obtained from the height difference of the upper and lower boundaries. Under the premise of stable atmospheric stratification, the Ozmidov scale $L_{O}$ [20,21] can be used as the maximum value of the turbulence scale, and the turbulence kinetic energy dissipation rate and turbulence diffusion coefficient can be correlated: $\varepsilon =L_{O}{}^{2}N_{s}{}^{3}$, $K=\beta L_{O}{}^{2}N_{s}$, where β is a constant [10], and $N_{s}$ is the positive buoyancy frequency calculated from the sorted potential temperature profile. Suppose c is an empirical constant. Then, the turbulence kinetic energy dissipation rate and turbulence diffusion coefficient are related to the Thorpe length:

(2) $$\varepsilon =C_{k}L_{T}{}^{2}N_{s}{}^{3}$$

(3) $$K=\gamma \varepsilon N_{s}$$

where $C_{k}=c^{2}$. Here we set $C_{k}$ = 0.3 [13] and γ = 0.25 [12].

## 3. Removal of Instrument Noise

The turbulent region identified by the Thorpe method is the region where the potential temperature decreases with height. These regions are defined as "inversion" regions, which include the "overturn" caused by real turbulent motion and artificial "inversion" caused by instrument noise. In Wilson’s study, it was pointed out that for the detection results of low-resolution (5–9 m) balloon data, only 7.9% of potential temperature "inversion" data were selected as real turbulent "overturn" data, and the rest of the "inversion" was considered to be noise interference and removed [18]. Therefore, when using the Thorpe method for turbulence retrieval, noise removal is particularly critical. In order to ensure the reliability of the results, it is necessary to choose a scientific and effective denoising method.

### 3.1. Instrument Noise

**Figure 2:**
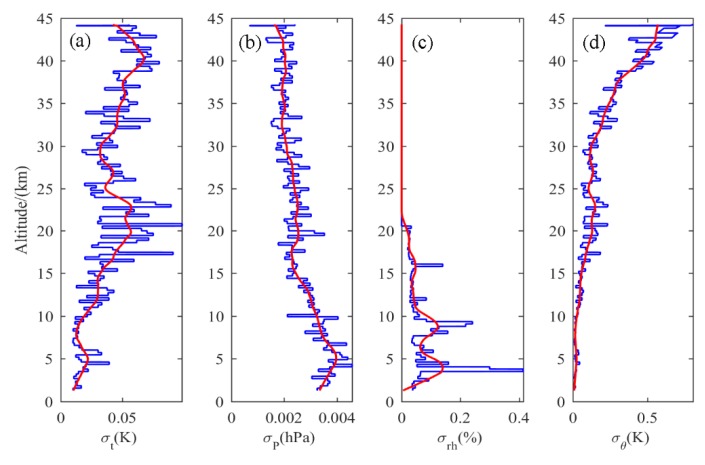
Standard deviations of noise on **(a)** temperature, **(b)** air pressure, **(c)** relative humidity, and **(d)** potential temperature; the red curve represents the smoothed value.

While the sensor carried by the radiosonde receives the temperature, pressure, and humidity data, the unwanted signal caused by the instrument is also involved; that is, the instrument noise. According to the study of Gavrilov et al. [10], the structure function method can be used to estimate the instrument noise variance. The steps for estimating the noise are as follows: First, the entire profile is split into segments about 200 m in length (in this paper, 30 bins are selected as a segment, and the interval is 210 m), and the trend is obtained by linear fitting on each segment. Then, the residual can be obtained by subtracting the trend from the original signal, and the instrument noise variance is half of the variance of the first difference of the residual data. Finally, the noise level can be expressed as the root mean square of the noise variance, which is the noise standard deviation [18]. Taking the balloon data from the night of September 21 as an example, the noise standard deviations of temperature ($\sigma_{t}$), air pressure($\sigma_{P}$), relative humidity ($\sigma_{rh}$), and potential temperature ($\sigma_{\theta }$) are calculated by the above process, and the results are shown in Figure 2. The potential temperature is calculated by the temperature and pressure, so the noise standard deviation of the potential temperature can be obtained by the error transfer formula [18]:

(4) $$\sigma_{\theta }=\theta \sqrt{{\left(\frac{\sigma_{t}}{T}\right)}^{2}+{\left(\frac{2}{7}\frac{\sigma_{P}}{P}\right)}^{2}}$$

As can be seen from Figure 2, $\sigma_{t}$ is basically within 0.1 K of the whole detection height range. The relatively large noise of the temperature sensor around 20 and 40 km may be caused by the violent pendulum motion of the radiosonde due to strong wind shear. The $\sigma_{P}$ value is less than 0.4 Pa and tends to decrease with altitude. The $\sigma_{rh}$ is less than 0.4% and reaches its maximum at 5 km. After that, the $\sigma_{rh}$ decreases rapidly with height and is basically 0 when the height is over 23 km. Compared to the temperature sensors, the humidity sensors and pressure sensors are less susceptible to pendulum movement due to their higher sensitivity and smaller hysteresis coefficients. However, $\sigma_{\theta }$ increases with height, especially for noise levels above 35 km. It can be seen from Equation (4) that the degree of interference of potential temperature by noise is related to the noise of both the pressure sensor and the temperature sensor. Since the temperature noise is an order of magnitude larger than the pressure noise for the whole height, ${\left(\frac{\sigma_{t}}{T}\right)}^{2}$ is much larger than ${\left(\frac{2}{7}\frac{\sigma_{P}}{P}\right)}^{2}$. The calculation formula for potential temperature noise can be simplified as $\sigma_{\theta }\approx \theta \frac{\sigma_{T}}{T}={\left(\frac{1000}{P}\right)}^{2/7}\sigma_{T}$. The pressure decreases faster as the height increases, corresponding to the rapid increase in noise on potential temperature. Notably, above 35 km, although the sensor noise remains at a low level, the accuracy of turbulence detection will be affected due to the rapidly increasing potential temperature noise.

### 3.2. Denoising Method

**Figure 3:**
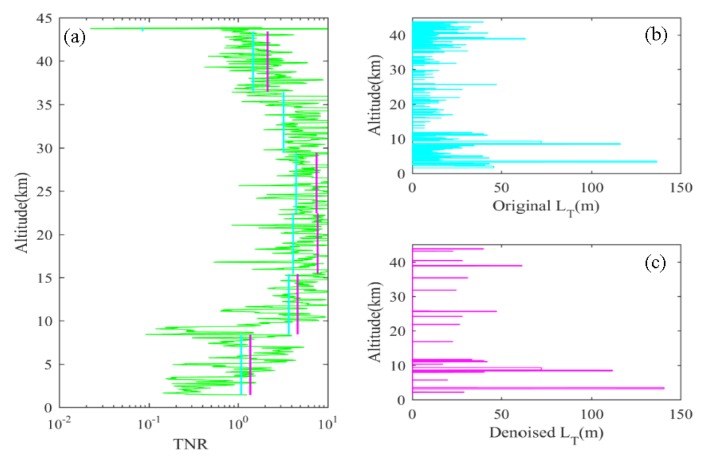
(**a**) The vertical distribution of trend-to-noise ratio (tnr) (cyan solid line: bulk tnr, purple solid line: averaged local tnr), (**b**) Thorpe length before denoising, (**c**) Thorpe length after denoising.

A large part of the inversion of the potential temperature profile is caused by the interference of instrument noise. For a monotonically increasing potential temperature profile, whether an artificial inversion will occur after noise is added depends on the atmospheric stratification state, vertical resolution, and noise level. In order to further measure the atmospheric level of instrument noise, the concept of trend-to-noise ratio (tnr) is introduced here [22]. The overall tnr within a range is defined as

(5) $$\bar{\zeta }=\frac{\theta_{n}-\theta_{1}}{{\bar{\sigma }}_{\theta }(n-1)}$$

where n is the number of sample bins in an interval, and ${\bar{\sigma }}_{\theta }$ is the average of the standard deviation of the potential temperature noise in the interval. When $\bar{\zeta }$ is less than 1.5, the atmosphere has a weak stratification stability, and artificial inversion is easily generated. Here, the Thorpe analysis is seriously disturbed by the noise and cannot determine the upper and lower boundary of the inversion, which requires smoothing and under-sampling processes. The smoothed and under-sampled window m at this data resolution is taken as 2; here, reference is made to [18] for the processing of low-resolution profiles. Meanwhile, the local noise ratio is defined as

(6) $$\zeta_{i}=\frac{{\theta^{\prime }}_{i+1}-{\theta^{\prime }}_{i-1}}{2\sigma_{i}}$$

where $\theta^{\prime }$ is the reordered potential temperature profile, and $\sigma_{i}$ is the noise level of the ith point. The variation of tnr with height is shown in Figure 3a. The green curve represents the smoothed local trend-to-noise ratio; the whole profile is divided into several segments according to 7 km, and the overall trend-to-noise ratio and the corresponding average value of the local trend-to-noise ratio on each segment are calculated separately. The cyan solid line and the purple solid line are, respectively, used to represent the bulk tnr and the average value of the corresponding local noise-to-noise ratio for each segment (the average value of the local tnr on the fifth segment is too large and is not shown in the figure). It can be seen that the bulk tnr is small at 0–7 km and above 35 km, and the stratification stability is weak at 0–7 km due to strong convection, so the potential temperature profile is highly susceptible to noise. Above 35 km, although the stratification stability is strong, the noise of the potential temperature begins to increase significantly, so it is also prone to artificial inversion. Before applying the Thorpe method to these two intervals, smoothing and under-sampling were performed.

For the potential temperature profile processed above, we believe that the stratification stability of the whole height satisfies the premise of the Thorpe method. On this basis, the real turbulent patch is selected by comparing the inversion size caused by the noise simulated by the Monte Carlo simulation method with the real calculated overturn size [22]. The hypothesis is as follows: if the number of sample points in an inversion is *n*, the potential temperature range of the overturn caused by the turbulence is $W_{\theta }$. When the number of sampling points *n* is determined, *n* variables of normal distribution are obtained. W is the difference between the maximum value and the minimum value of variables. A total of 1000 sets of W are obtained by the Monte Carlo simulation. $W_{N}(n)$ is selected from the noise ranges W(i) (i = 1,2,3, …,1000) simulated by Monte Carlo and satisfies the following relationship: $\mathrm{Pr}[W<W_{N}(n)]=N/100$ (here, we set subscript N to 95, corresponding to the significance level α=0.05). The noise standard deviation of the inversion is $\sigma_{n}$. When $W_{\theta }>\sigma_{n}W_{N}$, the inversion is selected as a real turbulent overturn.

Figure 3b (Figure 3c) shows the distribution of the Thorpe length with height before (after) denoising. Before denoising, there are 226 inversion regions, including both the artificial inversion caused by the noise and the real turbulent overturns. There are 22 real turbulent layers selected after denoising, and only 9.7% of the inversion regions are considered to be turbulent layers and retained, which is similar to the results in [18].

## 4. Results and Discussion

There are three main mechanisms for the generation of turbulence in the atmosphere. One is the convective turbulence generated by the static instability of the atmosphere. The energy source of the convective turbulence is obtained directly or indirectly through buoyancy work. The second is the mechanical turbulence caused by wind shear. Turbulent kinetic energy is obtained by the shear stress work. The third is the turbulence caused by the waves. The increase of wind shear makes the amplitude of a gravity wave increase continuously. When the amplitude increases to a certain extent, turbulence will be generated due to fragmentation. Using the data obtained from the sounding data, the background stratification stability of the atmosphere is reflected by the wind speed, buoyancy frequency, wind shear, and Richardson number.

Buoyancy frequency can be used to measure the motion state of air particles after a disturbance in stable stratification. When $N^{2}>0$, the disturbed air particle oscillates and remains in a static, stable state. When $N^{2}<0$, the particle is disturbed by convection, which is a static unstable state. Here, the relative threshold method [23] is adopted to judge whether the air water vapor is saturated, and then the buoyancy frequency under unsaturated water vapor $N_{d}{}^{2}$ [24] and buoyancy under saturated water vapor $N_{m}{}^{2}$ [25] are obtained, respectively:

(7) $$N_{d}{}^{2}=\frac{g}{T}\left[\left(\frac{\partial T}{\partial Z}\right)+\Gamma_{d}\right]$$

(8) $$N_{m}{}^{2}=\frac{g}{T}\left[\left(\frac{\partial T}{\partial Z}\right)+\Gamma_{m}\right]\left[1+\frac{L_{v}q_{s}}{RT}\right]-\frac{g}{1+q_{w}}\left(\frac{dq_{w}}{dz}\right)$$

where $\Gamma_{d}$ is the dry adiabatic decline rate; $\Gamma_{m}$ is the wet saturation decline rate; $L_{v}$ is the latent heat of the evaporation of liquid water or ice; $q_{w}=q_{L}+q_{s}$, where $q_{L}$ is the mixture ratio of liquid water or ice; and $q_{s}$ is the saturated mixture ratio.

The gradient Richardson number Ri can reflect the ratio of buoyancy work and shear stress work, which can be characterized by

(9) $$Ri=\frac{\bar{N^{2}}}{{\left(\frac{\partial \bar{u}}{\partial z}\right)}^{2}+{\left(\frac{\partial \bar{v}}{\partial z}\right)}^{2}}$$

**Figure 4:**
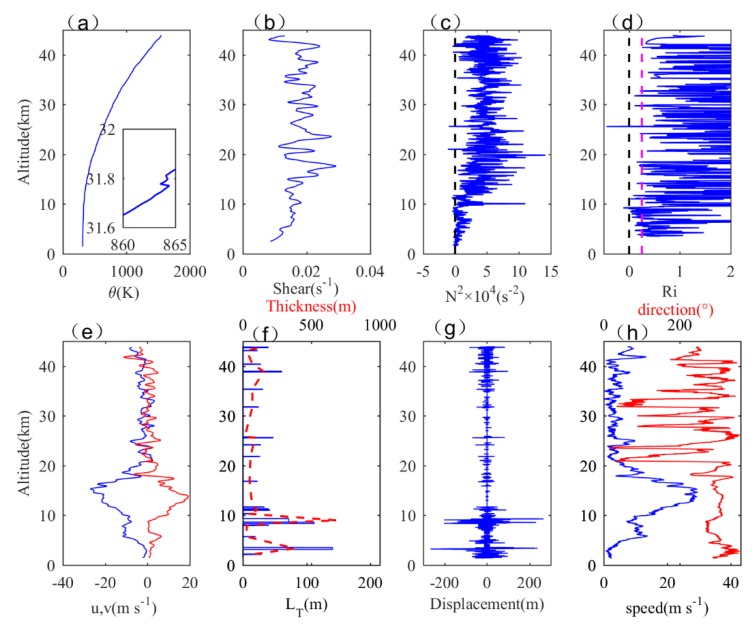
Vertical distribution of turbulence parameters at 19:00 on September 21, 2019: (**a**) potential temperature (θ), (**b**) vertical shear of wind speed (shear), (**c**) square buoyancy frequency (N^2^), (**d**) gradient Richardson number (purple dotted line: Ri = 0.25), (**e**) wind speed component (blue curve: zonal component u; red curve: meridional component v), (**f**) Thorpe length LT (blue solid line) and turbulent layer thickness (red dotted line), (**g**) Thorpe displacement, and (**h**) synthetic wind speed (blue curve) and wind direction (red curve).

Considering the influences of small-scale fluctuations and noises, the wind speed ($\bar{u}$,$\bar{v}$) is averaged at an interval of 35 m, and $\bar{N^{2}}$ is averaged at an interval of 70 m. In this paper, data on the evening of September 21, 2019 are selected first. Figure 4a–h shows the vertical distribution of the potential temperature profile, wind shear, buoyancy frequency, gradient Richardson number, meridional and zonal wind components, Thorpe length, Thorpe displacement, wind speed, and wind direction, respectively. As can be seen from Figure 4a, the overall potential temperature profile shows an increasing trend with height but includes many small inversions, which can be clearly seen through the enlarged rectangular box. In Figure 4b, the maximum value of wind shear occurs near 17 and 23 km, with values of 0.0290 and 0.0276 s^−1^, respectively, and there is an area with a significant increase in wind shear above 13 km. In Figure 4c, the buoyancy frequency below 12 km is relatively low, and many regions are less than 0, indicating that the atmospheric stratification is unstable, and convective activity occurs relatively easily, whereas the buoyancy frequency above 12 km significantly increased, with almost no negative value, and the atmospheric stratification became stable. Figure 4d shows the distribution of the gradient Richardson number; the purple dotted line represents Ri = 0.25, and the area with Ri <0.25 is also concentrated below 12 km, indicating that the troposphere is more likely to generate turbulence. In the stratosphere, Ri is almost greater than 0.25, indicating that the turbulence generated in the stratosphere can correspond to a larger Richardson number. In Figure 4e, the blue curve represents the zonal wind, and the red curve represents the meridional wind. The westerly jet is dominant over Hami, so the zonal wind is significantly greater than the meridional wind. As can be seen from Figure 4f, the turbulence layers obtained are more densely distributed in the troposphere, while in the stratosphere, they are sparsely distributed. The intensity of turbulence is also smaller in the stratosphere than that in the troposphere. Moreover, the variation trends of the Thorpe length, turbulent layer thickness, and Thorpe displacement are basically consistent. The jet stream area above the tropopause is about 13–14 km^2^, and the wind direction near the jet stream area is stable at around 300°. In the troposphere, there are many unstable regions where buoyancy and turbulence function and occur more easily with stronger intensity. In the stratosphere, the stratification stability of the atmospheric is strong. Here, turbulence is mainly caused by wind shear and the saturation and fragmentation process during the upward propagation of gravity waves, and the distribution of turbulence in this area is sparse with weaker intensity.

**Figure 5:**
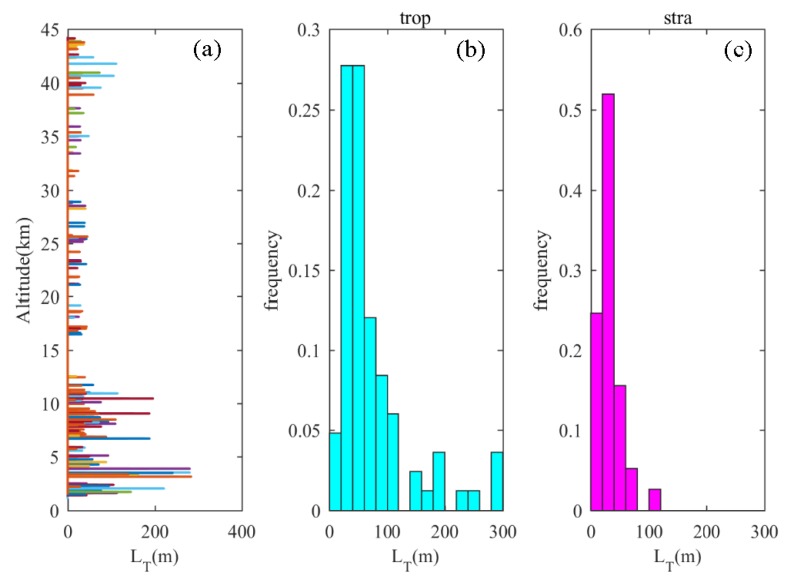
(**a**) Vertical distribution of the Thorpe length, (**b**) frequency distribution of the tropospheric Thorpe length, (**c**) frequency distribution of the stratospheric Thorpe length.

The above results verify the good applicability of the Thorpe method to the near-space high-resolution balloon data. All nine groups of data were thus used to retrieve the turbulence and calculate its parameters, and a total of 166 turbulence layers were obtained. The cumulative distribution result of the Thorpe length is shown in Figure 5. The largest Thorpe length is concentrated in the troposphere, and the distribution of Thorpe length in the stratosphere is scattered and less intense. In the troposphere, 38.9% of the Thorpe length is greater than 60 m, while in the stratosphere, the proportion falls to 9.2%. The Thorpe length in the troposphere can reach a maximum of 300 m, whereas in the stratosphere, it can only reach 120 m. The buoyancy frequency in the stratosphere is positive, the atmosphere is stable, and the vertical exchange of air particles in the upper and lower layers is more difficult. Therefore, the resulting Thorpe displacement is smaller and the turbulence mixing intensity is weaker. In the troposphere, the buoyancy frequency is close to zero, and there are many regions with a negative buoyancy frequency, corresponding to a weaker stratification stability. Although filtering and undersampling have been used to enhance stratification stability before applying Thorpe’s method, the influence of thermal convection cannot be ignored. Thus, the part of the troposphere with a large Thorpe length is subjected to both shear stress and buoyancy.

**Figure 6:**
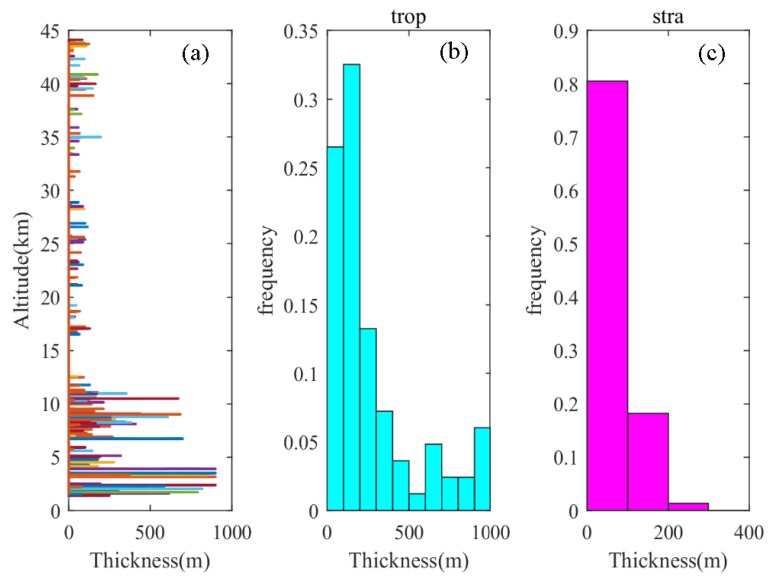
(**a**) Vertical distribution of turbulence thickness, (**b**) frequency distribution of tropospheric turbulence thickness, (**c**) frequency distribution of stratospheric turbulence thickness.

The Thorpe length can reflect the intensity of turbulent mixing, while the turbulence thickness can reflect the vertical scale of the detected turbulent layer. Figure 6 shows the cumulative distribution of turbulent layer thickness. It can be seen from Figure 6a that the distribution trend of turbulence thickness is basically consistent with the Thorpe length. According to the principle of sorting by the Thorpe method (inside the uniformly mixed turbulent layer), in the thicker turbulent layer, there are more air particles with larger Thorpe displacements; the Thorpe length in this layer is the root mean square of the internal Thorpe displacements, so the turbulent layer with greater thickness usually corresponds to stronger turbulence intensity. In the troposphere, the maximum turbulence thickness can reach 1000 m, while in the stratosphere, it can only reach 300 m. The turbulent layer with a thickness greater than 100 m accounts for 71.43% in the troposphere, while in the stratosphere, it is concentrated within 100 m, and the turbulent layer with a thickness greater than 100 m is only 23%. In the troposphere, the thickness of the turbulent layer is generally larger, so it can be well identified by the radiosonde. In the stratosphere, the vertical scale of turbulence is generally smaller, and most of the minor turbulence with a vertical scale smaller than the resolution of the radiosonde cannot be identified, resulting in the sparse distribution of the stratospheric turbulence obtained by the Thorpe method.

**Figure 7:**
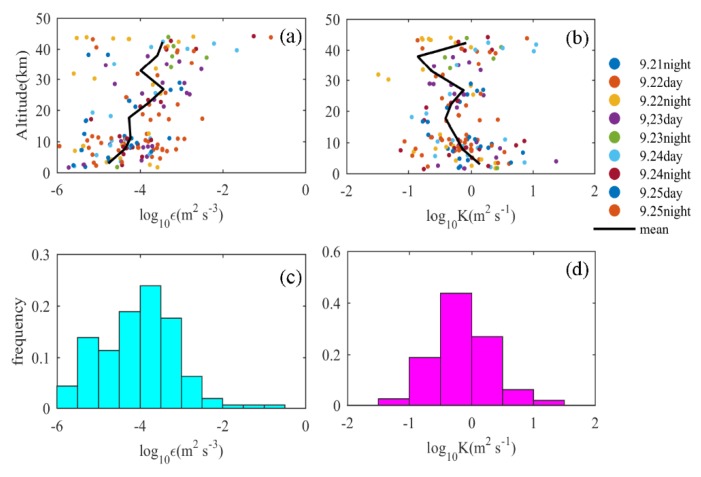
(**a**) Vertical distribution of the turbulent dynamic energy dissipation rate, (**b**) vertical distribution of turbulent diffusion coefficient, (**c**) frequency distribution of the turbulent dynamic energy dissipation rate, (**d**) frequency distribution of the turbulent diffusion coefficient; the black curve is the profile after averaging at 5 km vertical intervals.

The turbulence kinetic energy dissipation rate and turbulence diffusion coefficient can effectively describe the effectiveness of turbulence mixing and provide an important reference for the study of atmospheric physical and chemical processes. Therefore, it is necessary to have a clear and intuitive understanding of these two parameters. Figure 7 shows the distribution of the turbulence kinetic energy dissipation rate ε and the turbulence diffusion coefficient K, which is calculated from formula (2) and (3). The magnitude of ε is between 10^−6^ and 10^0^ m^2^s^−3^ and is mainly concentrated between 10^−6^ and 10^−2^ m^2^s^−3^, accounting for 95.18%. The magnitude distribution of K is between 10^−2^ and 10^2^ m^2^s^−1^, mainly focused between 10^−1^ and 10^1^ m^2^s^−1^, accounting for 92.17%. In order to obtain more details of the vertical distribution, ε and K were averaged over an interval of 5 km, and the black curve represents the average values of ε and K. ε increases with height, while K first decreases with height and then increases. Values of ε below 10 km have a relatively concentrated distribution. The distribution range of ε is about two orders of magnitude. However, above 10 km, the distribution of ε become progressively more disperse as its height increases, and the distribution range of ε at 40 km reaches five orders of magnitude. For K, the distribution range over the whole height is within two orders of magnitude. It can be seen from formula (2) and (3) that ε is related to $L_{T}{}^{2}$ and $N^{3}$, while K is related to $L_{T}{}^{2}$ and N. In the troposphere, the value of N is relatively small, and the distribution is concentrated, while in the stratosphere, the value of N is significantly increased, and the fluctuation is more obvious, with a wider distribution range. The influence of the $N^{3}$ term on the value of ε is greater than that of the $L_{T}{}^{2}$ term; in contrast, the influence of the N term on the value of K is less than that of the $L_{T}{}^{2}$ term. The buoyancy frequency increases with height, and the Thorpe length decreases with height. Therefore, the ε is affected by $N^{3}$ and tends to increase with height, while K is affected by $L_{T}{}^{2}$ and tends to decrease with height. Combined with the above parameters, it can be found that in an atmosphere with stable stratification, large-scale turbulence has strong turbulent diffusion and weak dynamic energy dissipation, while in a stratified stable atmosphere, small-scale turbulence has weak turbulent diffusion and strong dynamic energy dissipation.

**Figure 8:**
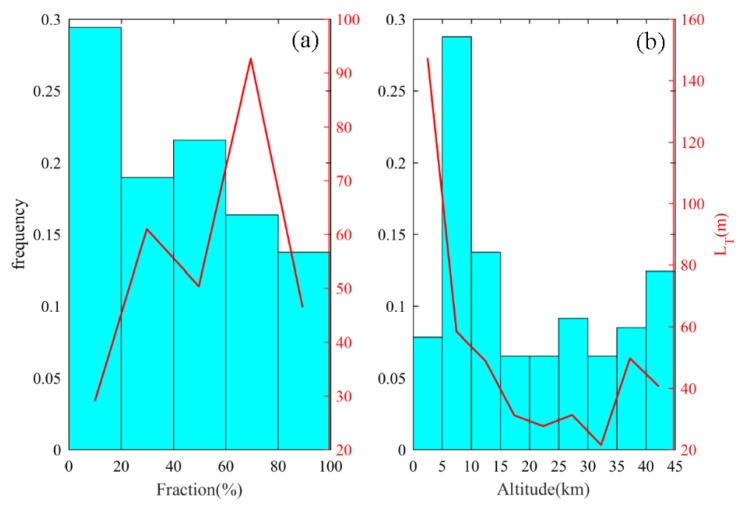
(**a**) The frequency distribution of the turbulent fraction (cyan column) and the average Thorpe length (red curve) of the corresponding distribution interval; (**b**) the frequency distribution of the turbulence (cyan columnar) at different height intervals; and the average Thorpe length (red curve) of the corresponding distribution interval.

In order to further explore the relationship between the internal characteristics of turbulence and the turbulence parameters, here we define the turbulence fraction F, which is the proportion of the area with Ri < 0.25 in an independent turbulence layer. The greater the value of F, the stronger the turbulent mixing degree and the greater the internal instability. Figure 8a shows the frequency distribution for the turbulent fraction. F is divided according to the ranges of [0%, 20%), [20%, 40%), [40%, 60%), [60%, 80%), and [80%, 100%], and the Thorpe length in each interval is averaged and represented by a red curve. Figure 8b shows the frequency distribution of turbulence at different height intervals with an interval of 5 km, and the average Thorpe length at each interval is represented by a red curve. It can be seen that F has the largest amount of turbulence at an interval of [0, 20%), accounting for 28.48%, while the amount of turbulence is the lowest at an interval of [80%, 100%], with only 13.91%. At the interval of [0, 20%), the average Thorpe length is the smallest at only 29 m, and the average Thorpe length at the interval of [20%, 60%] is approximately 50–60 m; the average Thorpe length reached a maximum of 92.4 m in [60%, 80%). When F is between [80%, 100%], the average Thorpe length decreased significantly, and the mean $L_{T}$ was only 46.5 m. We found that with an increase of the turbulent fraction, the turbulent mixing effect was gradually strengthened, and the corresponding turbulence intensity also increased. However, when turbulent mixing reached a critical value, the internal instability continued to increase, and the turbulent layer could not continue to be maintained and began to decay. The region with the strongest turbulence intensity is between 0 and 5 km. With an increase of height, the turbulence intensity decreases significantly. Above 35 km, the turbulence intensity increases. The turbulence at a low altitude is affected by thermal convection. The stratification instability of the atmosphere promotes the formation and development of turbulence, so the intensity is strong. The turbulence at a high altitude is mainly caused by wind shear, which occurs in a stable atmosphere. The upper and lower exchanges are suppressed, so the turbulence is weak. The turbulence intensity above 35 km increases abnormally, as it is obviously affected by noise. As shown in Figure 2d, the noise of potential temperature starts to increase significantly above 35 km. Small turbulence disturbances are easily disturbed by noise signals. The Thorpe method cannot distinguish between small turbulence disturbances and large noise signals, resulting in a larger turbulent layer thickness with a stronger Thorpe length. Thus, the intensity of turbulence will increase significantly above 35 km. 

**Figure 9:**
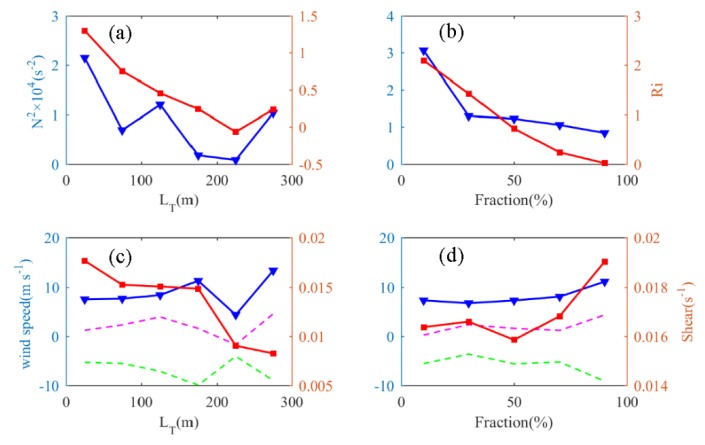
(**a**) The variation of buoyancy frequency and Richardson number with Thorpe length (blue curve: buoyancy frequency, red curve: Richardson number), (**b**) the variation of buoyancy frequency and Richardson number with turbulence fraction, (**c**) the variation of wind speed and wind shear with Thorpe length (blue curve: synthetic wind speed, red curve: wind shear, green dashed line: zonal wind component, purple dashed line: meridional wind component), (**d**) the variation of wind speed, and wind shear with turbulent fraction.

Parameters like buoyancy frequency, Richardson number, wind shear, and wind speed may affect the occurrence and development of turbulence. Thus, we calculated the corresponding parameters of each turbulent layer (average) and explored the correlations between them. All the turbulent layers are grouped according to an interval of 50 m for the Thorpe length. Buoyancy frequency and the Richardson number of the layers in each interval are averaged, as shown in Figure 9a. The values of the wind speed, wind shear, zonal wind component, and meridional wind component are averaged, and the results are shown in Figure 9c. All turbulent layers are grouped according to an interval of 20% of the turbulence fraction. The variation trend of the buoyancy frequency and Richardson number with the turbulent fraction is shown in Figure 9b, and the variation trends of wind speed, wind shear, zonal wind component, and meridional wind component with the turbulent fraction are shown in Figure 9d. With an increase of turbulence intensity, both the Richardson number and buoyancy frequency decrease, but the Richardson number and buoyancy frequency of the region with the largest turbulence intensity remain near zero and do not reach the minimum value. With a gradual increase of the turbulent fraction, the buoyancy frequency and Richardson number are gradually reduced; the turbulent mixing becomes greater and more functional as the atmospheric stratification is weakened.

It can be seen from Figure 8 that the turbulence intensity is largest when F is between 60% and 80%, but the Richardson number and the buoyancy frequency do not reach the minimum value, which is consistent with the situation that the maximum value of $L_{T}$ does not correspond to the minimum value of Ri and $N^{2}$ in Figure 9. This indicates that a weaker layer stability is conducive to the generation of turbulence; however, when the unstable energy is greater than a certain degree, the turbulence cannot continue to be maintained and begins to decay, and the turbulence intensity decreases. For wind speed, the variation trends of the zonal wind component and meridional wind component are basically the same. That is, the two increase synchronously or decrease synchronously. In the turbulent layer with a Thorpe length between 200 and 250 m, the average wind speed is the smallest—only 4.4 m/s. These turbulence layers also correspond to the minimum buoyancy frequency and Richardson number, which indicates that when the atmospheric stratification is unstable, the vertical transport of the atmosphere is relatively strong, making it difficult to form a stable and sustained strong wind band. These turbulences are mainly dominated by convective turbulence. Wind speed has little effect on the whole process of turbulent mixing, and wind speed changes little with the turbulent fraction. For wind shear, it can be seen that as the turbulence intensity increases, the wind shear gradually decreases, and the wind shear inside the turbulent layer with a Thorpe length larger than 200 m is within 0.01 s^−1^. With an increase of the turbulent fraction, the average wind shear decreases first and then increases, indicating that wind shear can cause turbulence, but strong wind shear may disrupt the structure of turbulence, which is not conducive to the maintenance and development of turbulence.

## 5. Main Conclusions

Using the Thorpe method, this paper analyzed near-space, high-resolution balloon sounding data for Hami, Xinjiang from September 21 to 25, 2019. The process of turbulence retrieval and noise removal was introduced in detail. The distribution characteristics are discussed, and the relationships between the development process of turbulence and atmospheric background parameters were explored.

The turbulent energy dissipation rate is more susceptible to stratification stability of the atmosphere, and the value of ε increases with height. Generally, the turbulent diffusion coefficient is more susceptible to turbulence intensity, and the value of K decreases first and then increases. The generation mechanism of turbulence is explored in combination with the atmospheric background state. The result shows that in a stratified, unstable atmosphere, turbulence with a large scale experiences strong turbulence diffusion and weak turbulence kinetic energy dissipation. However, in a stratified stable atmosphere, turbulence with a small scale experiences weak turbulence diffusion and strong turbulence kinetic energy dissipation. The two areas where turbulence activity is relatively strong are 3–4 and 8–11 km. Given the Tianshan mountains and the generally hilly topography in Hami, the strong turbulence in the lower troposphere is mainly caused by terrain waves and thermal convection, and the significantly enhanced turbulence near the tropopause could thus be attributed to the breaking of Kelvin–Helmholtz waves [26] and enhanced dynamic instability. Above 11 km, as static stability increases rapidly, mechanical turbulence can only exist when the shear stress does enough work to overcome the damping effect of stability, which leads to significantly weakened turbulence in the stratosphere.

Since the degree of material exchange during the whole process of turbulence is different, the concept of turbulence fraction is used to describe the degree of turbulence mixing. With an increase in the turbulence fraction, the turbulence mixing effect is gradually strengthened, corresponding to an increase in turbulence intensity. However, when the turbulence mixing reached its critical value, the internal instability continued to increase; thus, the turbulence layer could not be maintained, and the turbulence intensity decreased. The results of this paper show that turbulence intensity reaches its maximum value when the turbulence fraction is between 60% and 80% and starts to decrease when the turbulence intensity exceeds 80%. Both the Richardson number and buoyancy frequency decreased with an increase in the turbulence fraction, while wind shear increased with an increase of the turbulence fraction. The average wind speed hardly affected the mixing degree inside the turbulent layer.

By analyzing the sensor noise, it was found that the effect of the Thorpe method in analyzing turbulence is limited by height. This type of radiosonde data can provide the complete detection results of turbulence between 0 and 45 km. The use of radiosonde data for the analysis of atmospheric disturbances, such as turbulence and gravity, to a higher altitude, will help improve our understanding of the characteristics of the atmospheric temperature and dynamic structure in the near space [27]. In our results, the amount of turbulence after denoising is less than 10% of all potential temperature inversions. Turbulence over 35 km clearly begins to be interfered with by noise from potential temperature, and small-scale turbulence is easily confused with noise signals, resulting in a larger Thorpe length and thickness for the obtained turbulence. Although we can roughly determine the general position and intensity of turbulence at these heights, the turbulence parameters cannot be obtained accurately. We believe that for this type of radiosonde, detection accuracy is mainly affected by the rapid reduction of atmospheric pressure above a certain height because the noise levels of the temperature, pressure, and humidity sensors are kept at a reasonable range, although the temperature sensor is more susceptible to the interference of pendulum motion and increases measurement errors. However, after eliminating the interference of the noise on the results, this type of sensor can accurately detect the turbulence below 35 km in Northwest China, which is currently the highest height achieved by the Thorpe method for turbulence detection using radiosonde data.

In view of the problem that the Thorpe method is limited by height for detecting turbulence, an improved denoising method is needed to distinguish small-scale turbulence and noise signals, which will be the next step in our future work.

## Figures and Tables

**Table 1:** The accuracy of sensors (T—temperature; P—pressure; RH—relative humidity; G—wind direction; w—wind velocity).

Parameter	Measuring Range	Accuracy
T	50 ~−90 °C	40~50 °C: ΔT ≤ ±0.2 °C −80~40 °C: ΔT ≤ ±0.2 °C −90~−80 °C: ΔT ≤ ±0.3 °C
P	1060 hPa~5 hPa	5 hP~200 hPa:ΔP ≤ ±0.7 hPa 200~1060 hPa:ΔP ≤ ±1.5 hPa
RH	0%~100% RH	15%~95% RH: ΔRH ≤ ±3% RH
G	0°~360°	ΔG ≤ ±3°
w	0~150 m/s	Δw ≤ ± 0.2 m/s

## References

[1] Lubken F.J., Hillert W., Lehmacher G., Von Zahn U. (1993). Experiments revealing small impact of turbulence on the energy budget of the mesosphere and lower thermosphere. J. Geophys. Res..

[2] Lübken F.J. (1997). Seasonal variation of turbulent energy dissipation rates at high latitudes as determined by in situ measurements of neutral density fluctuations. J. Geophys. Res. Atmos..

[3] Hocking W.K. (1983). On the extraction of atmospheric turbulence parameters from radar backscatter doppler spectra-I. Theory. J. Atmos. Terr. Phys..

[4] Hocking W.K. (1999). The dynamical parameters of turbulence theory as they apply to middle atmosphere studies. Earth Planets Space.

[5] Rüster R., Klostermeyer J. (1987). Propagation of turbulence structures detected by VHF radar. J. Atmos. Terr. Phys..

[6] Haack A., Gerding M., Lübken F.J. (2014). Characteristics of stratospheric turbulent layers measured by LITOS and their relation to the Richardson number. J. Geophys. Res..

[7] Lolli S., Delaval A., Loth C., Garnier A., Flamant P.H. (2013). 0.355-micrometer direct detection wind lidar under testing during a field campaign in consideration of ESA’s ADM-Aeolus mission. Atmos. Meas. Tech..

[8] Schneider A., Gerding M., Lübken F.J. (2015). Comparing turbulent parameters obtained from LITOS and radiosonde measurements. Atmos. Chem. Phys..

[9] Thorpe S.A. (1977). Turbulence and mixing in a scottish loch. Philos. Trans. R. Soc. London Ser. A.

[10] Gavrilov N.M., Luce H., Crochet M., Dalaudier F., Fukao S. (2005). Turbulence parameter estimations from high-resolution balloon temperature measurements of the MUTSI-2000 campaign. Ann. Geophys..

[11] Luce H., Fukao S., Dalaudier F., Crochet M. (2002). Strong mixing events observed near the tropopause with the MU radar and high-resolution balloon techniques. J. Atmos. Sci..

[12] Clayson C.A., Kantha L. (2008). On turbulence and mixing in the free atmosphere inferred from high-resolution soundings. J. Atmos. Oceanic Technol..

[13] Nath D., Ratnam M.V., Patra A.K., Murthy B.V.K., Rao S.V.B. (2010). Turbulence characteristics over tropical station Gadanki (13.5°N,79.2°E) estimated using high-resolution GPS radiosonde data. J. Geophys. Res..

[14] Satheesan K. (2002). Turbulence parameters in the tropical troposphere and lower stratosphere. J. Geophys. Res..

[15] Zhang J., Zhang S.D., Huang C.M., Huang K.M., Gong Y., Gan Q., Zhang Y.H. (2019). Statistical study of atmospheric turbulence by thorpe analysis. J. Geophys. Res. Atmos..

[16] Kohma M., Sato K., Tomikawa Y., Nishimura K., Sato T. (2019). Estimate of turbulent energy dissipation rate from the VHF radar and radiosonde observations in the antarctic. J. Geophys. Res. Atmos..

[17] Luce H. (2014). Simultaneous observations of tropospheric turbulence from radiosondes using Thorpe analysis and the VHF MU radar. Radio Sci..

[18] Wilson R., Dalaudier F., Luce H. (2011). Can one detect small-scale turbulence from standard meteorological radiosondes?. Atmos. Meas. Tech..

[19] Thorpe S.A. (2005). The Turbulent Ocean.

[20] Li D., Salesky S.T., Banerjee T. (2016). Connections between the Ozmidov scale and mean velocity profile in stably stratified atmospheric surface layers. J. Fluid Mech..

[21] Smyth W.D., Moum J.N., Caldwell D.R. (2001). The efficiency of mixing in turbulent patches: Inferences from direct simulations and microstructure observations. J. Phys. Oceanogr..

[22] Wilson R., Luce H., Dalaudier F., Lefrère J. (2010). Turbulence patch identification in potential density or temperature profiles. J. Atmos. Oceanic Technol..

[23] Zhang J., Chen H., Li Z., Fan X., Peng L., Yu Y., Cribb M. (2010). Analysis of cloud layer structure in Shouxian, China using RS92 radiosonde aided by 95 GHz cloud radar. J. Geophys. Res. Atmos..

[24] Emanuel K.A., Hide R. (2008). Atmospheric convection. Phys. Today.

[25] Durran D.R., Klemp J.B. (2002). On the effects of moisture on the brunt-väisälä frequency. J. Atmos. Sci..

[26] Browning K.A., Bryant G.W., Starr J.R., Axford D.N. (1973). Air motion within Kelvin-Helmholtz billows determined from simultaneous Doppler radar and aircraft measurements. Q. J. R. Meteorol. Soc..

[27] Zhao X.R., Sheng Z., Li J.W., Yu H., Wei K.J. (2019). Determination of the “wave turbopause” using a numerical differentiation method. J. Geophys. Res. Atmos..

